# Fusion, rupture, and degeneration: the fate of *in vivo*-labelled PSVs in developing barley endosperm^*^

**DOI:** 10.1093/jxb/eru175

**Published:** 2014-05-06

**Authors:** Verena Ibl, Eszter Kapusi, Elsa Arcalis, Yasushi Kawagoe, Eva Stoger

**Affiliations:** ^1^Department for Applied Genetics and Cell Biology, Molecular Plant Physiology and Crop Biotechnology, University of Natural Resources and Life Sciences, Muthgasse 18, 1190 Vienna, Austria; ^2^Division of Plant Sciences, National Institute of Agrobiological Sciences, Tsukuba, 305–8602Japan

**Keywords:** Cereal seed, endomembrane system, endosperm, protein storage vacuole, TIP3.

## Abstract

Cereal endosperm is a highly differentiated tissue containing specialized organelles for the accumulation of storage proteins. The endosperm of barley contains hordeins, which are ultimately deposited within protein storage vacuoles (PSVs). These organelles have been characterized predominantly by the histochemical analysis of fixed immature tissue samples. However, little is known about the fate of PSVs during barley endosperm development, and *in vivo* imaging has not been attempted in order to gain further insight. In this report, young seeds were followed through development to characterize the dynamic morphology of PSVs from aleurone, subaleurone, and central starchy endosperm cells. TIP3-GFP was used as a PSV membrane marker and several fluorescent tracers were used to identify membranes and monitor endomembrane organelles in real time. Whereas the spherical appearance of strongly labelled TIP3-GFP PSVs in the aleurone remained constant, those in the subaleurone and central starchy endosperm underwent substantial morphological changes. Fusion and rupture events were observed in the subaleurone, and internal membranes derived from both the tonoplast and endoplasmic reticulum were identified within these PSVs. TIP3-GFP-labelled PSVs in the starchy endosperm cells underwent a dramatic reduction in size, so that finally the protein bodies were tightly enclosed. Potential desiccation-related membrane-altering processes that may be causally linked to these dynamic endomembrane events in the barley endosperm are discussed.

## Summary

The article provides *in vivo* imaging data on the morphological changes in the endomembrane architecture of developing barley seeds, focussing on the fate of protein storage vacuoles along endosperm development.

## Introduction

After differentiation, the fully developed endosperm can account for up to 75% of the seed weight and functions as a storage tissue that accumulates starch and storage proteins. These provide the nutrients required for germination and seedling growth until autotrophy is possible (Olsen, 2004). The endosperm comprises four major cell types: the outer epidermis-like aleurone cells, central starchy endosperm cells, transfer cells, and cells of the embryo-surrounding region (Olsen, 2001). Barley has three layers of aleurone cells (Olsen, 2004) which support germination by mobilizing starch and storage protein reserves in the starchy endosperm by releasing hydrolytic enzymes (Olsen, 2004). The starchy endosperm comprises subaleurone and central starchy endosperm cells. The subaleurone is located just beneath the aleurone and contains large quantities of storage proteins but only a few small starch granules, whereas the central starchy endosperm contains large and abundant starch granules and comparatively little protein (Bechtel and Pomeranz, 1978).

Seeds produce three major classes of storage proteins: prolamins, albumins, and globulins (Shewry *et al.*, 1995; Galili, 2004). Prolamins are the major protein component of most cereal seeds, and the Triticeae prolamin superfamily is divided into three groups: sulphur-rich, sulphur-poor and high-molecular-weight (HMW) prolamins. Similarly, three groups of prolamins are found in rice and maize, although in the latter species they are described as α, (β, γ), and δ zeins (Woo *et al.*, 2001). Barley prolamins include the sulphur-rich B and γ hordeins, the sulphur-poor C hordein, and the HMW D hordein, which is homologous to the HMW glutenins of wheat (Kreis and Shewry, 1989).

Prolamins and the soluble albumins and globulins follow different subcellular trafficking routes. Whereas albumins and globulins typically pass through the Golgi body and accumulate in the protein storage vacuole (PSV), most prolamins aggregate in the endoplasmic reticulum (ER) and are initially deposited into ER-derived protein bodies, which may also be incorporated into the PSV in some cereal species (Shewry *et al.*, 1995; Galili, 2004; Tosi *et al*., 2009*a*,*b*). Hordein precursor polypeptides are cotranslationally inserted into the ER lumen and finally deposited in the vacuole (Cameron-Mills and Wettstein, 1980; Rechinger *et al.*, 1993). Although there is no direct evidence for their transport through the Golgi body, this route cannot be excluded, at least for part of the hordein content of the seed. Alternatively, hordein polypeptides may exit directly from the ER and be taken up by the PSV in an autophagy-like process, as suggested for wheat prolamins (Levanony *et al.*, 1992).

Tonoplast intrinsic proteins (TIPs) are widely used as tonoplast markers for vacuolar biogenesis and identity in higher plants. TIPs are a subfamily of aquaporins and form channels that facilitate the movement of water, small uncharged solutes, and gases (Kaldenhoff and Fischer, 2006; Maurel, 2007). TIP3 (α-TIP) was first characterized as TP25, a seed-specific aquaporin that is strongly expressed in seeds but decreases rapidly during germination (Johnson *et al.*, 1989). TIP3 is synthesized on the rough ER and appears to reach the tonoplast without passing through the Golgi body (Johnson *et al.*, 1989; Hillmer *et al.*, 2001; Gattolin *et al.*, 2010). Recent expression assays and microscopy in *Arabidopsis*, barley aleurone protoplasts, and pea cotyledons confirmed that TIP3 is predominantly associated with PSVs in the seeds (Paris *et al.*, 1996; Schuurink *et al.*, 1996; Swanson *et al.*, 1998; Jauh *et al.*, 1999; Jiang and Rogers, 1999; Hunter *et al.*, 2007; Gattolin *et al.*, 2009, 2010, 2011), and TIP3 has recently been used as a PSV (PBII) marker in rice subaleurone cells (Onda *et al.*, 2009).

Given the technical dexterity required to access endosperm tissue and study the subcellular structures in cereal seeds, most information concerning the subcellular composition of seed tissue and characterization of protein storage organelles and their contents has been derived from static images (Craig *et al.*, 1980; Shewry and Halford, 2002; Arcalis *et al*., 2004, 2010; Holding *et al.*, 2007). Histochemical studies of barley endosperm midway through development have identified hordein-containing PSVs in the subaleurone layer (Rechinger *et al.*, 1993). However, little is known about the fate of PSVs during cereal endosperm development.

In this investigation, early stage seeds were followed through development to characterize the morphological changes of PSVs in different cell layers. Because fixed samples do not fully reveal the dynamic endomembrane processes in the ER and vacuolar compartments, TIP3-GFP was selected as a PSV membrane marker and used together with other fluorescent endomembrane tracers to monitor dynamic endomembrane processes in real time. The recombinant proteins OsTIP3::TIP3-GFP and OsTIP3::Cherry-SEC61 were combined with the fluorescent markers MDY-64 and ER-Tracker to visualize the vacuolar and ER membranes. Confocal laser scanning microscopy (CLSM) then allowed the simultaneous morphological characterization of PSVs in aleurone, subaleurone, and starchy endosperm cells and revealed cell-specific changes in PSV morphology as well as restructuring events during endosperm development. Potential stress-related mechanisms that occur during desiccation and that may facilitate these dynamic endomembrane events in the barley endosperm are discussed.

## Materials and methods

### Barley cultivation

Barley plants (wild-type variety Golden Promise and its transgenic derivative TIP3-GFP) were grown in incubation rooms under a 16/8 light/dark cycle (16 °C and 70% relative humidity) for 2 months. After tillering, cultivation was continued at 22 °C.

### Cloning OsTIP3::CherrySEC61

Plasmid DNA was isolated from *Escherichia coli* 214–5 and 331–3, containing the vectors pTIP3::TIP3dsRED and pAPS2::CherrySEC61, respectively (Onda *et al.*, 2009). CherrySEC61 was transferred to pTIP3::TIP3dsRED by replacing TIP3dsRED using *Kpn*I and *Sac*I restriction sites. The final construct (pTIP3::CherrySEC61) was verified by sequencing.

### Transforming barley with OsTIP3::TIP3-GFP and OsTIP3::CherrySEC61

Immature barley embryos were used as explants for particle bombardment (Wan and Lemaux, 1994) with the fluorescent marker constructs plus vector p6U containing the hygromycin phosphotransferase (*hpt*) selection marker (DNA Cloning Service, Hamburg, Germany). Transgenic callus was grown on callus induction medium (Hensel *et al.*, 2009) containing 50mg l^–1^ hygromycin B (Roche, Mannheim, Germany), then on plant regeneration medium containing 25mg l^–1^ hygromycin B. Small plantlets were tested by PCR for the presence of *hpt* and the fluorescent marker constructs. Positive plantlets were transferred to climate chambers for self-pollination and seed set.

### Preparation for CLSM

For immunofluorescence analysis, homozygous T_4_ seeds from transgenic TIP3-GFP plants (17 days after pollination, DAP) were fixed in 4% (w/v) paraformaldehyde for 3h and then in 2% (w/v) paraformaldehyde for 12h. After washing with 0.1M phosphate buffer (pH 7.4), 60–100 μm vibratome sections were prepared and placed on glass slides coated with 0.1% (w/v) polylysine (Sigma). Sections were dehydrated through an ethanol series and equilibrated in 0.1M phosphate buffer. The cell wall was digested with 2% (w/v) cellulase (Onozuka R10 from *Trichoderma viride*) in phosphate buffer (0.1M, pH 7.4) for 1h at room temperature. Following treatment with 0.5% Triton X-100 in phosphate buffer for 1h at room temperature, nonspecific binding sites were blocked with 3% (w/v) bovine serum albumin (fraction V) in phosphate-buffered saline (0.14M NaCl, 0.0027M KCl, 0.01M PO_4_, pH 7.4). for 10min. The sections were then incubated with a monoclonal antibody against GFP diluted 1:100 in phosphate buffer overnight at 4 °C. Antibody binding was visualized using a goat anti-rabbit IgG (H+L) conjugated to Alexa Fluor 488 (Life Technologies). The sections were mounted in 50% glycerol in phosphate buffer and observed by CLSM.

For fluorescence imaging without immunodetection, transgenic TIP3-GFP barley seeds were sectioned, washed, and mounted in tap water and analysed by CLSM. Wild-type Columbia *Arabidopsis* seeds and the transgenic line GFP-TM-KKXX (Benghezal *et al.*, 2000) were soaked in water for at least 2h at 4 °C before the seed coats were removed. Wild-type seed was incubated with 2 μM ER-Tracker Green (diluted from 1mM DMSO stock with tap water) for 1h at 22 °C, mounted in tap water and observed by CLSM. GFP-TM-KKXX seed was mounted in tap water without treatment and observed directly by CLSM. Transgenic CherrySEC61 barley seeds were chipped and incubated with 2 μM ER-Tracker Green for 1h at 22 °C, then sectioned, mounted in tap water, and observed by CLSM. For *in vivo* time lapse experiments, 10 images were captured per minute with a line average of 16.

### CLSM

Images were captured using the Leica SP5 CLSM with filter settings for autofluorescence (excitation wavelength 405nm, emission wavelength 410–480nm), GFP (excitation 488nm, emission 500–530nm), ER-Tracker Red (excitation 561nm, emission 571–623nm), ER-Tracker Green (excitation 488nm, emission 500–531nm), and Alexa 488 (excitation 488nm, emission 503–544nm). Images were processed using Leica confocal software version 2.61, ImageJ and Adobe Photoshop CS5.

### Calculation of compartment sizes

The diameter of starch granules was calculated by measuring the length of the major axes of the ellipse: n=108, 129, and 27 for 8, 10, and 12 DAP, respectively. The size of the TIP3-GFP compartment was calculated by measuring the maximum distance between opposite membranes: n= 114, 168, and 35 for 8, 10, and 12 DAP, respectively.

### Transmission electron microscopy

Developing seeds were harvested and bisected longitudinally allowing the embryo to be removed. Thin slices were cut from the endosperm with a razor blade under 0.1M phosphate buffer (pH 7.4) and fixed in 4% (w/v) paraformaldehyde plus 0.5% (v/v) glutaraldehyde in phosphate buffer overnight at 4 °C. Sections showing silver interference colours were collected on gold grids, contrasted, and observed using a FEI Tecnai G^2^ transmission electron microscope as previously described (Arcalis *et al.*, 2010).

## Results

### TIP3-GFP-labelled PSVs were predominantly detected in the aleurone and subaleurone of TIP3-GFP transgenic barley endosperm

OsTIP3::TIP3-GFP was used to study the vacuolar dynamics of the developing barley endosperm because it was recently described as a PSV (PBII) marker in rice subaleurone cells (Onda *et al.*, 2009). Transgenic barley lines were generated by the bombardment of immature embryos with particles coated in the corresponding expression construct and the presence of the transgene in the regenerated plants was confirmed by PCR. Seeds midway through development were taken from the middle of the ears of eight transgenic plants and were prepared for *in vivo* CLSM.

**Fig. 1.**
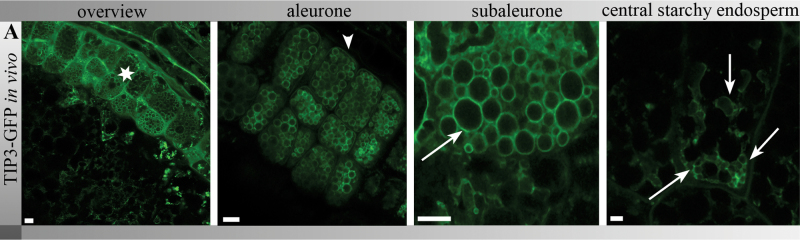
TIP3-GFP localized predominantly to PSVs in mid-mature aleurone and subaleurone cells of barley endosperm (*in vivo* imaging). Note the strong signal in the aleurone (asterisk), weak labelling of TIP3-GFP at the plasma membrane (arrowhead), and PSVs in the subaleurone and central starchy endosperm (arrows). Bars, 5 μm. Corresponding bright-field images are provided in Supplementary Fig. S1.

TIP3-GFP was strongly expressed in the barley aleurone and, to a lesser extent, in the subaleurone and central starchy endosperm (Fig. 1, asterisk), labelling the tonoplast of PSVs and, to a lesser extent, the plasma membrane (Fig. 1, arrowhead). These results confirmed previous studies in transgenic *Arabidopsis* cotyledon cells (where TIP3-GFP was localized on the tonoplast of PSVs and on the plasma membrane) and immunofluorescence data from barley aleurone protoplasts labelled with α-TIP (Schuurink *et al.*, 1996; Gattolin *et al.*, 2011). In the subaleurone, TIP3-GFP labelling was mainly restricted to spherical membrane compartments resembling PSVs (Fig. 1, arrow), whereas in the central starchy endosperm, TIP3-GFP labelled smaller PSVs with irregular shape (Fig. 1, arrows). Notably the TIP3-GFP signal was much weaker in the central starchy endosperm compared to the subaleurone and aleurone (Fig. 1). Corresponding bright-field images are provided in Supplementary Fig. S1A (available at *JXB* online).

Because live cell imaging is hampered in maturing seeds by the accumulation of starch, further histochemical studies of TIP3-GFP transgenic seeds were carried out using an anti-GFP antibody. Consistent with the *in vivo* data, immunofluorescence microscopy confirmed the localization pattern of TIP3-GFP in the aleurone, subaleurone, and central starchy endsosperm of seeds at 17 DAP (Supplementary Fig. S1B, asterisk). In the aleurone, TIP3-GFP was again localized on the tonoplast of PSVs and on the plasma membrane (Supplementary Fig. S1B, arrowheads). In contrast to the spherical TIP3-GFP PSVs in the subaleurone, irregularly shaped PSVs labelled with TIP3-GFP were observed in the central starchy endosperm (Supplementary Fig. S1B, arrow). The TIP3-GFP signal was again weaker in the central starchy endosperm than in the aleurone and subaleurone.

The ultrastructure of fixed wild-type barley seeds stained with toluidine blue was also used to characterize the morphology of barley endosperm (Supplementary Fig. S1C). Spherical PSVs were detected in the aleurone, confirming the spherical appearance of aleurone TIP3-specific PSVs in the earlier experiments (Supplementary Fig. S1C, asterisk). Because toluidine blue stains proteins, these experiments confirmed the high storage protein content in the subaleurone compared to the starchy endosperm (Supplementary Fig. S1C). Furthermore, small putative vacuolar structures were observed in the starchy endosperm (Supplementary Fig. S1C, arrows).

Taken together, the *in vivo* and histochemical data indicated that PSVs were predominantly found in the aleurone and subaleurone. Simultaneous *in vivo* analysis of TIP3-GFP-labelled PSVs in aleurone, subaleurone, and central starchy endosperm cells revealed that TIP3-GFP-labelled PSVs were predominantly spherical compartments in the aleurone and subaleurone but showed a smaller, irregular shape and fainter labelling in the central starchy endosperm.

### TIP3-GFP-labelled PSVs underwent morphological changes during endosperm development

**Fig. 2.**
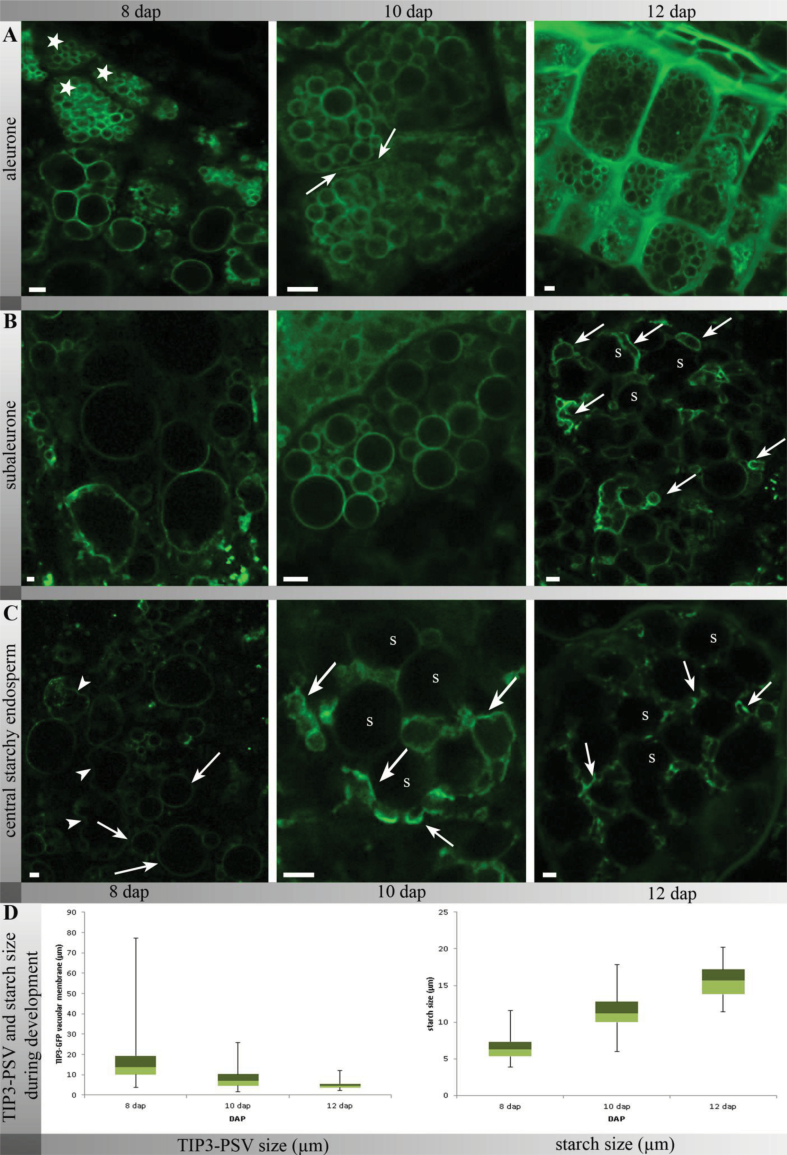
TIP3-GFP-labelled PSVs underwent morphological changes during endosperm development. In the aleurone (A), TIP3-GFP labelled PSV membranes strongly (asterisk) and the plasma membrane weakly (arrows) at 8–12 DAP. In subaleurone (B) and starchy endosperm cells (C), TIP3-GFP appeared first at spherical (arrows) and irregularly shaped (arrowheads) membrane compartments (arrows) that changed in shape and size during development, resulting in small compartments with discontinuous membranes at 12 DAP in the central starchy endosperm (arrows); bars, 5 μm; s, starch; corresponding bright-field images are provided in Supplementary Fig. S2. (D) Descriptive box plot statistics of the developmental size changes of TIP3-GFP-labelled PSVs and starch granules in the subaleurone and central starchy endosperm. Values are mean, interquartile range, and minimum and maximum.

In order to characterize the morphological changes of PSVs during barley endosperm development, the aleurone, subaleurone, and starchy endosperm of 8, 10, 12, 17, and 21-DAP-old transgenic TIP3-GFP seeds were imaged by CLSM (Fig. 2). The most dramatic morphological changes were observed between 8 and 12 DAP.

In differentiated aleurone cells, TIP3-GFP was localized on the membrane of small, spherical compartments representing PSVs (Fig. 2A, 8 DAP, asterisk). During subsequent development, TIP3-GFP continued to highlight spherical PSVs in aleurone cells as well as weak staining of the plasma membrane (Fig. 2A, arrows).

In the subaleurone at 8 DAP, TIP3-GFP was localized to large spherical vacuolar compartments, although these became smaller by 10 DAP. Starch granules become more prominent during maturation, so most of the TIP3-GFP-labelled PSVs became smaller and less regular in shape by 12 DAP (Fig. 2B, arrows), and no further visible changes occurred during further seed development.

In the central starchy endosperm at 8 DAP, TIP3-GFP was localized at spherical (Fig. 2C, 8 DAP, arrows) and at irregularly shaped membrane compartments (Fig. 2C, 8 DAP, arrowheads). As in the subaleurone, the TIP3-GFP-labelled compartments became smaller during development. At 10 DAP, small TIP3-GFP-labelled PSVs were observed between the starch granules, and at 12 DAP, small and unevenly shaped TIP3-GFP-labelled PSVs were tightly packed between starch granules (Fig. 2C). At 12 DAP, many PSVs appeared to lack continuous TIP3-GFP-labelled membranes (Fig. 2C) and this tendency continued during subsequent maturation, as will be discussed further in detail.

This study quantified the size reduction of TIP3-GFP-labelled membrane compartments and compared it with the growth of starch granules in subaleurone and central starchy endosperm cells at 8, 10, and 12 DAP, as shown by box plots representing the descriptive statistics of the large dataset (Fig. 2D). The diameter of TIP3-GFP-labelled PSVs was ~17.6 μm, rising to a maximum of 77.2 μm at 8 DAP, but this fell to ~7.97 μm (maximum 25.9 μm) at 10 DAP and ~4.92 μm (maximum 11.9 μm) at 12 DAP. In parallel, starch granule size increased from ~6.45 μm at 8 DAP to ~11.45 μm at 10 DAP and ~15.60 μm at 12 DAP. The size of the TIP3-GFP-labelled vacuolar compartments was thus reduced by ~33% between 8 and 10 DAP, whereas the starch granules grew by ~40% over the same duration.

In summary, the simultaneous analysis of the size and morphology of TIP3-GFP-labelled PSVs in the aleurone, subaleurone, and central starchy endosperm *in vivo* during endosperm development showed that the structures remained morphologically stable in the aleurone but underwent significant changes in the subaleurone and central starchy endosperm, confirming that the fate of TIP3-GFP-labelled PSVs was dependent on the cell layer.

### TIP3-GFP-labelled PSVs in the barley subaleurone contained ER-membrane-associated protein bodies, small ER-derived structures, and TIP3-GFP-positive membrane fragments

It is well documented that hordein polypeptides are synthesized in the ER lumen of barley subaleurone cells and ultimately deposited as protein bodies within the PSVs, although the transport route has yet to be defined (Cameron-Mills and Wettstein, 1980). To characterize the process in more detail, subaleurone cells in TIP3-GFP transgenic seeds were labelled with ER-Tracker and analysed by *in vivo* CLSM. The fidelity of ER-Tracker in plants was confirmed by staining *Arabidopsis* seeds expressing a fluorescent ER marker and by staining transgenic barley seeds expressing mCherry-SEC61 (data not shown).

**Fig. 3.**
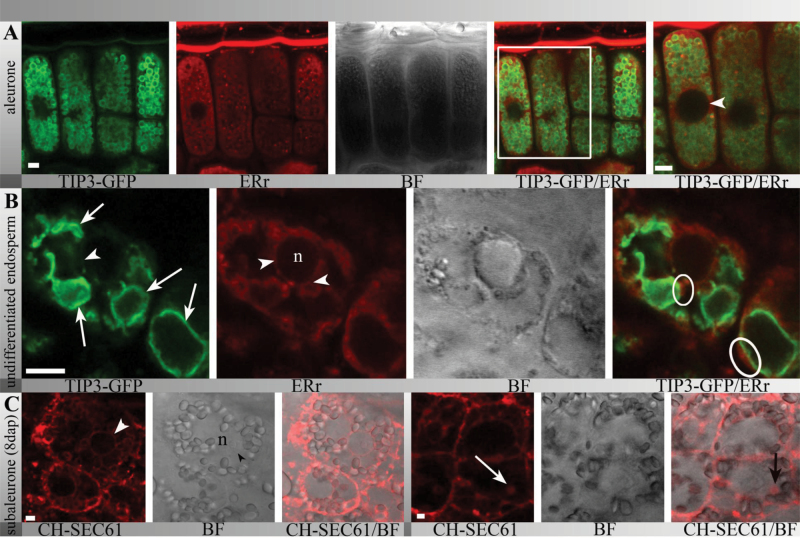
Confocal laser scanning microscopy showing the simultaneous localization of TIP3-GFP and the ER network in barley endosperm. (A) In the aleurone, CH-SEC61 localized to the ER network including the nuclear envelope (arrowhead) and was distinct from TIP3-GFP-labelled PSVs; red spherical signal within TIP3-GFP-positive PSVs represents the autofluorescence of the globoid inclusion. (B) In young undifferentiated endosperm (<8 DAP), TIP3-GFP already strongly labelled PSVs in close proximity to the ER network labelled with ER-Tracker Red (circles), including the nuclear envelope (arrowhead). (C) Internal endosperm at 8 DAP showed that CH-SEC61 was localized on the nuclear envelope (arrowhead) and small spherical structures, possibly protein bodies (arrow). Bars, 5 μm.

**Fig. 4.**
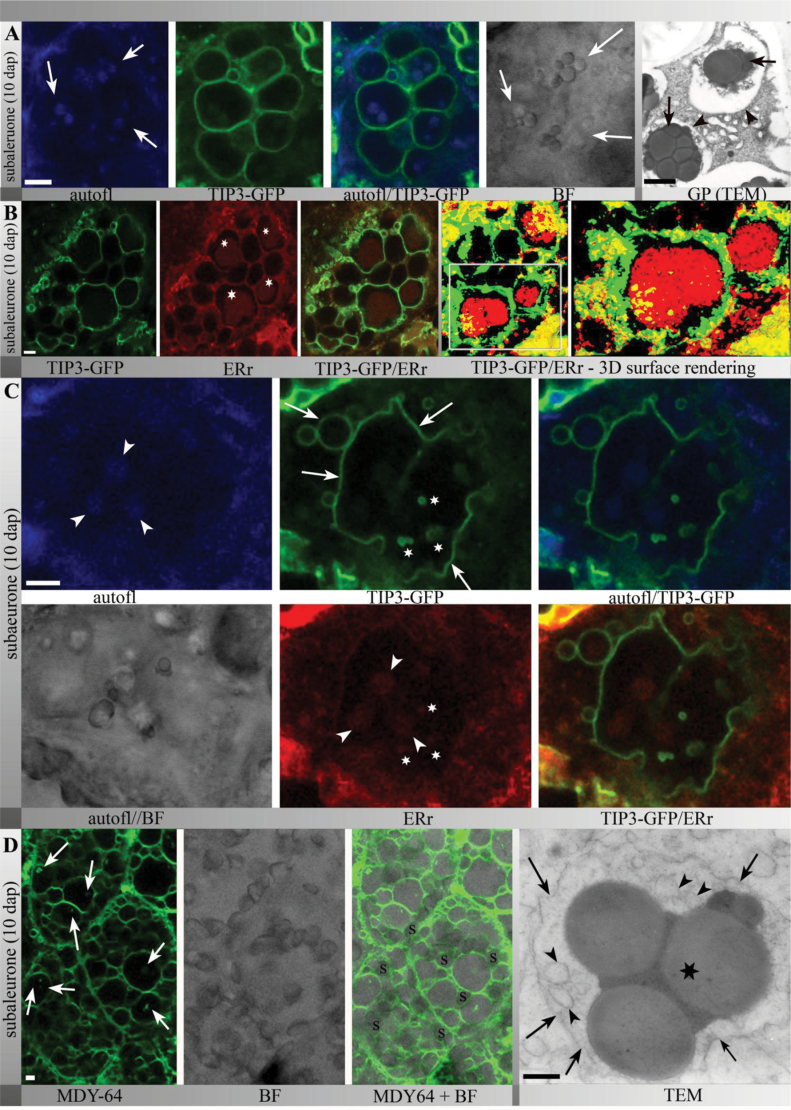
(A) The PSVs of subaleurone cells (10 DAP) in TIP3-GFP transgenic seeds were strongly labelled and contained autofluorescent protein bodies (arrows); note the corresponding protein body structures in the bright-field image; transmission electron micrographs also showed protein bodies (arrows) within PSVs (arrowhead) in the subaleurone. (B) TIP3-GFP-labelled PSVs comprised large protein bodies stained with ER-Tracker Red (asterisks); see also the three-dimensional surface rendering of 16 sections with a step size of 0.5 μm. (C) ER-Tracker labelling colocalized with autofluorescent protein bodies (arrowhead) that were deposited within large and small TIP3-GFP-labelled PSVs (arrows); note the presence of small TIP3-GFP-labelled vesicles enclosing structures labelled with ER-Tracker Red (asterisks) within large TIP3-GFP-specific vacuoles. (D) Intravacuolar membranes in sections stained with the strong vacuolar membrane marker MDY-64 revealed vacuolar membranes (arrows) within vacuolar compartments; corresponding transmission electron micrographs showed protein bodies (asterisk) within a membrane compartment (arrows); note the small vesicles (arrowheads) lying close to the protein body. Bars, 5 μm for CLSM and 1 μm for TEM.

Aleurone tissue from TIP3-GFP lines (12 DAP) was then stained with ER-Tracker and this revealed clear labelling of the ER network between the TIP3-GFP-labelled PSVs and at the nuclear envelope (Fig. 3A, arrowhead). Similarly, in the earlier undifferentiated endosperm, the TIP3-GFP signal was already localized to the vacuolar membranes (Fig. 3B, arrows), whereas ER-Tracker highlighted the ER network and the nuclear envelope (Fig. 3B, arrowheads). Interestingly, the ER network and vacuolar structures were in close proximity (Fig. 3B, circles). At 8 DAP, ER-Tracker highlighted the ER network including the nuclear envelope in the subaleurone of transgenic plants expressing mCherry-SEC61 (Fig. 3C, arrowhead). Furthermore, mCherry-SEC61 highlighted punctate structures (Fig. 3C, arrow), possibly representing protein bodies. After the onset of storage protein deposition, strong autofluorescence was observed within the TIP3-GFP-labelled PSVs at 10 DAP (Fig. 4A, arrows). The autofluorescence was clearly associated with protein bodies, which appeared as granular structures in bright-field images (Fig. 4A). Accordingly, transmission electron micrographs of the barley subaleurone revealed the presence of protein bodies (Fig. 4A) within vacuolar compartments (Fig. 4A, arrowheads).

Dual labelling showed that the protein bodies within TIP3-GFP-labelled PSVs were stained with ER-Tracker, indicating the presence of ER-derived membranes, perhaps delimiting individual protein bodies that cluster within the PSVs (Fig. 4A–C). Furthermore, these experiments revealed large and irregularly shaped TIP3-GFP-labelled PSVs (Fig. 4C, arrows) containing several autofluorescent protein bodies that were also stained with ER-Tracker (Fig. 4C, arrowheads). Notably, these large TIP3-GFP-labelled PSVs frequently contained additional TIP3-GFP-positive membranes enclosing small structures stained with ER-Tracker (Fig. 4C, asterisks).

To confirm the identity of the vacuolar membranes within large TIP3-GFP-labelled PSVs, the strong fluorescent vacuolar membrane stain MDY-64 was used to highlight intravacuolar membrane compartments (Abrahams *et al.*, 2003). To this end, sections of barley seeds (10 DAP) were stained with MDY-64 and analysed *in vivo* by CLSM. MDY-64 labelled the mobile membrane compartments within vacuoles intensely (Fig. 4D, arrows). Moreover, transmission electron micrographs of barley subaleurone cells clearly showed membrane vesicles and fragments (Fig. 4D, arrowheads) next to the protein bodies (Fig. 4D, asterisk) within the PSVs (Fig. 4D, arrows). These findings supported the *in vivo* observation of TIP3-GFP-labelled loose membranes and vesicles within TIP3-GFP-positive PSVs in the subaleurone.

Taken together, these data showed that TIP3-GFP-labelled PSVs in the subaleurone were morphologically diverse and comprised one or more ER membrane-associated protein bodies and additional internal TIP3-GFP-labelled membrane structures. The uneven shape of the PSVs, the changing size during development, and the presence of intravacuolar TIP3-GFP-positive membranes indicated that reshaping events must have taken place in the developing barley subaleurone.

### TIP3-GFP-labelled PSVs were involved in fusion and rupture processes

**Fig. 5.**
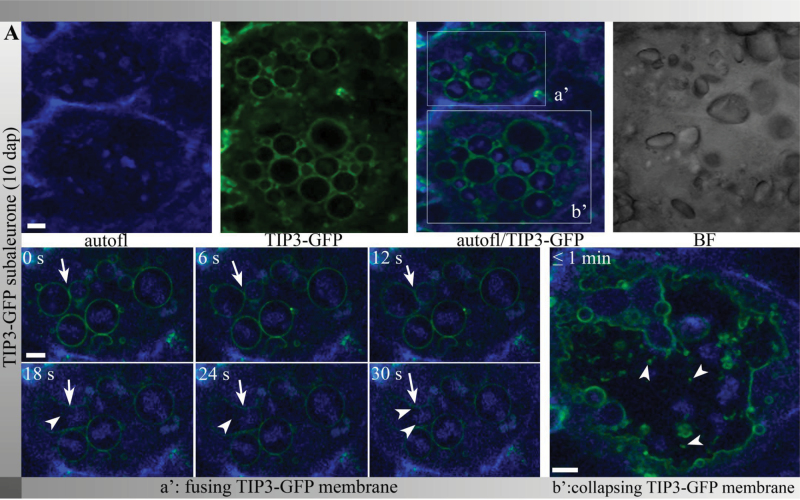
TIP3-GFP-labelled PSVs contained protein bodies and were involved in fusion and collapse processes. Live cell imaging of TIP3-GFP-labelled PSVs of fusion (a’) and collapse (b’) processes in the subaleurone at 10 DAP; images were acquired every 6 s. Note the presence of TIP3-GFP-labelled vesicles after fusion (a’, arrowheads). After the collapse of TIP3-GFP-labelled PSVs, TIP3-GFP vesicles and membranes were observed (b’, arrowheads). Interestingly, the protein bodies were not tightly enclosed by the TIP3-GFP-labelled membranes after collapsing. Bars, 5 μm. The corresponding movie is provided as supplementary material (Supplementary Movie M1 available at *JXB* online).

To gain insight into the dynamic behaviour of TIP3-GFP-labelled PSVs in the barley endosperm, the movement of TIP3-GFP-positive membrane structures was followed in a time-course experiment. A time series for TIP3-GFP-labelled PSVs containing protein bodies in two neighbouring cells is shown in Fig. 5. Membrane fusion events were observed (Fig. 5a’), where two small vacuoles surrounding protein bodies fused to form a larger TIP3-GFP-labelled PSV concomitantly with the fusion of small protein bodies into larger aggregates (Fig. 4B). Notably, small TIP3-GFP-labelled vesicles appeared immediately after the fusion processes (Fig. 5a’, arrowheads). Simultaneously, small vacuoles were also observed to rupture, causing the release of unconfined protein bodies as well as TIP3-GFP-labelled vesicles and membranes (Fig. 5b’, arrowheads). Such observations were made in more than 20 independent experiments, and PSVs from the nearby aleurone layers remained unaffected in all cases. In addition, different dynamic processes were often observed simultaneously within one section, so these observations were unlikely to represent methodological artefacts and were thus likely to contribute to the morphological changes of TIP3-GFP-labelled PSVs during endosperm development. The fusion of TIP3-GFP-labelled PSVs appeared to favour the aggregation of protein bodies into larger composite structures that became more common as the seeds matured.

### Small TIP3-GFP-labelled PSVs tightly enclosed protein bodies in central starchy endosperm after 12 DAP and then degenerated as the seeds mature

**Fig. 6.**
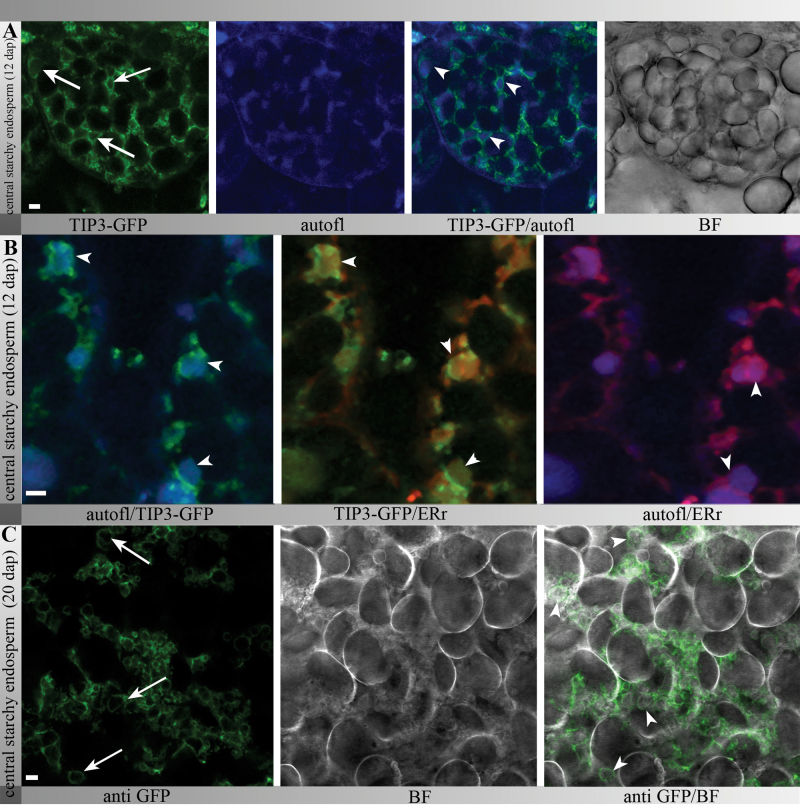
In advanced-stage central starchy endosperm cells, protein bodies were tightly enclosed by small TIP3-GFP-labelled PSVs. (A) CLSM *in vivo* studies at 12 DAP showed small TIP3-GFP-labelled PSVs (arrows) containing autofluorescent protein bodies (arrowheads). (B) Colocalization of TIP3-GFP, autofluorescent protein bodies, and ER-Tracker Red clearly showed autofluorescent protein bodies stained with ER-Tracker Red that were not continuously enclosed by TIP3-GFP-labelled membranes (C) Immunofluorescence analysis of TIP3-GFP central starchy endosperm cells at 20 DAP stained with an anti-GFP antibody showed small TIP3-GFP-labelled vacuoles (arrows) tightly surrounding protein bodies (arrowheads). Bars, 5 μm

Whereas the most dynamic reshaping events affecting TIP3-GFP-labelled PSVs occurred in the subaleurone, those in the central starchy endosperm also reduced in size. The simultaneous visualization of TIP3-GFP-labelled PSVs and autofluorescent protein bodies in the central starchy endosperm of transgenic seeds at 12 DAP revealed the presence of small, TIP3-GFP-labelled PSVs (Fig. 6A, arrows) tightly enclosing protein bodies (Fig. 6A, arrowheads). The simultaneous observation of TIP3-GFP-labelled PSVs, autofluorescent protein bodies, and ER-Tracker staining showed autofluorescent protein bodies surrounded by ER membranes and partially surrounded by TIP3-GFP-labelled membranes (Fig. 6B). Unlike in subaleurone cells, there was no evidence of a pronounced ER network in the central starchy endosperm at this developmental stage, indicating alterations of the ER membrane system.

To gain insight into the morphological changes at later developmental stages, sections of developing TIP3-GFP seeds at 20 DAP were labelled with an anti-GFP antibody. Single channel images of anti-GFP compared to bright-field again revealed that small TIP3-GFP-labelled PSVs (Fig. 6C, arrows) enclosed protein bodies (Fig. 6C, arrowheads) that fill the space between the starch granules. Transmission electron micrographs confirmed that the protein bodies were tightly enclosed by putative vacuolar membranes (Supplementary Fig. S2), supporting the aforementioned observations.

In summary, beyond 12 DAP, the central starchy endosperm of barley contained small TIP3-GFP-labelled PSVs that comprised autofluorescent and ER-Tracker positive protein bodies. Notably, the TIP3-GFP-labelled membranes did not always enclose the protein bodies continuously, perhaps indicating that the integrity of TIP3-GFP-labelled membranes declined during seed development. In mature seeds, the PSV membranes could still be observed by TIP3-GFP labelling and MDY-64 staining in aleurone and subaleurone, but were hardly detectable in the central starchy endosperm. In line with this, TIP3-GFP was detectable by immunoblot analysis of immature seeds, but not in samples from mature seeds (data not shown).

## Discussion

**Fig. 7.**
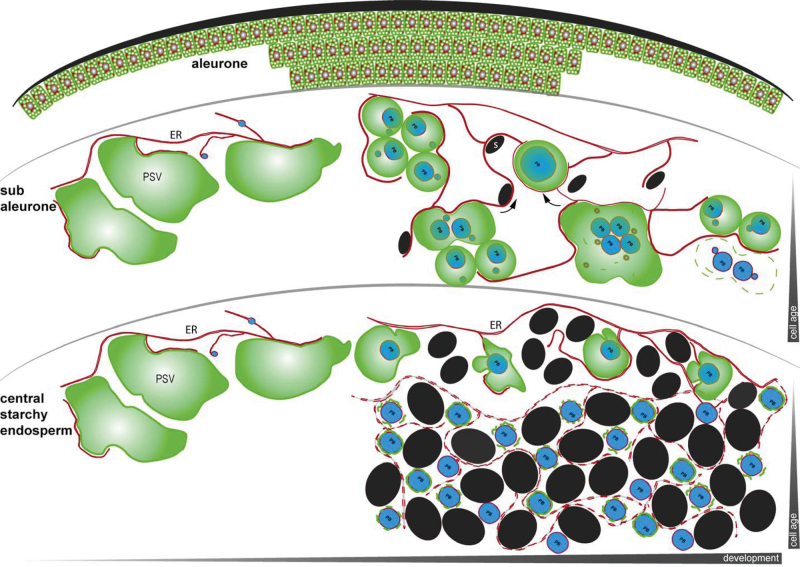
The fate of protein storage vacuoles in the aleurone, subaleurone, and central starchy endosperm of barley seeds. In the aleurone, small and spherical PSVs (green) remain apparently unchanged during endosperm development. The ER network including the nuclear envelope is indicated in red. In the subaleurone, large PSVs are present during early development, in intimate contact with the ER network. During endosperm development, PSVs become smaller, spherical structures enclosing protein bodies (blue), followed by fusion so that larger protein bodies are formed. Alternatively, PSVs may fuse and accumulate several protein bodies. Later, some PSV membranes collapse, releasing protein bodies without a tonoplast membrane. Only a few, small starch granules are present in the subaleurone. TIP3-GPF-labelled PSVs shrink during the development of both the subaleurone and central starchy endosperm, depending on the developmental stage and the cell age. However, PSVs do not appear to undergo fusion in the central starchy endosperm. At later developmental stages, protein bodies in the central starchy endosperm cells are tightly enclosed by PSV membranes, which degenerate during maturation, leaving protein bodies with no continuous PSV membrane or no membrane at all. Simultaneously, starch granules become more prominent in the starchy endosperm, and the ER membrane also degenerates at later developmental stages in this tissue (fragmented red ER signal). ER, endoplasmic reticulum; PSV, protein storage vacuole; S, starch.

Cereal endosperm cells and their storage compartments have been characterized mainly by histochemical fluorescence and electron microscopy studies using fixed samples at mid-maturation stages (Arcalis *et al.*, 2010; Tosi *et al.*, 2011; Reyes *et al.*, 2011). Although the fate of protein bodies within vacuoles is well characterized in the cereal subaleurone, little is known about the fate of PSVs and their membranes in developing cereal endosperm. Here, the dynamic morphology of TIP3-GFP-labelled PSVs was analysed in barley aleurone, subaleurone, and central starchy endosperm at different developmental time points, revealing cell-layer and stage-dependent morphology and behaviour, as summarized in Fig. 7. The intensity of the TIP3-GFP signal differed among aleurone, subaleurone, and starchy endosperm cells. The PSVs in the aleurone produced an intense signal, but this was progressively weaker in the subaleurone and starchy endosperm. This was unlikely to reflect the regulatory activity of the OsTIP3 promoter because another fluorescent fusion protein (mCherrySEC61), driven by the same promoter, was well expressed in barley starchy endosperm (data not shown). Furthermore, barley seeds older than 12 DAP stained with MDY-64 showed a similar distribution of fluorescence and also a stepwise decline in the signal from the aleurone to the starchy endosperm layers (data not shown). Therefore, the weak TIP3-GFP signal in starchy endosperm cells and its gradual decline during seed maturation indicated that there was a corresponding decline in the abundance of PSVs in the inner endosperm layers and a gradual loss of TIP3-GFP-labelled membranes. This is supported by the observation that protein bodies in the starchy endosperm during later development appeared only partially enclosed by TIP3-GFP-labelled membranes, suggesting that membrane integrity in the starchy endosperm may be affected during maturation.

The morphological changes of PSVs during endosperm development also differed among aleurone, subaleurone, and starchy endosperm cells (Fig. 7). Whereas TIP3-GFP-labelled PSVs in the aleurone did not change significantly, those in the other tissues reduced in size during development, particularly in the central starchy endosperm. The TIP3-GFP-labelled PSVs appeared as large compartments in both the subaleurone and central starchy endosperm at 8 DAP, but their morphology and behaviour differed during further seed development. In the subaleurone, they underwent fusion (allowing the protein bodies within to form larger, composite aggregates) and rupture (producing unconfined protein bodies and membrane fragments). Therefore, the TIP3-GFP-labelled PSVs at 10 DAP appeared as large, uneven compartments often containing several protein bodies, before shrinking during further seed maturation.

Occasionally, large TIP3-GFP-labelled PSVs containing large spherical protein bodies were still observed at advanced developmental stages in the subaleurone, possibly reflecting the presence of developmentally arrested cells. In contrast, no fusion events were observed in the central starchy endosperm cells, and the TIP3-GFP-labelled PSVs reduced their size considerably by 10 DAP so that the protein bodies were tightly enclosed, and the PSVs were eventually hemmed in by large starch granules. The pronounced morphological difference between subaleurone and central starchy endosperm cells may reflect their different origins, with the former derived from periclinal divisions of aleurone cells followed by redifferentiation (Tosi *et al.*, 2011). The morphology of the TIP3-GFP-labelled PSVs was influenced further by the age of individual cells. Thus, subaleurone cells just below the aleurone often differed from the remainder of the subaleurone, and outer starchy endosperm cells tended to differ from the central starchy endosperm, in both cases likely reflecting a centripetal increase of cell age (Fig. 7).

Interestingly, the labelling of membrane components revealed the presence of internal membranes in barley endosperm PSVs, such that protein body aggregates within the TIP3-GFP-labelled PSVs were marked by ER-Tracker, indicating that ER-derived membranes may be associated with individual and clustered protein bodies within subaleurone PSVs. This is in agreement with previous results obtained in wheat and maize (Levanony *et al.*, 1992; Reyes *et al.*, 2011) and suggests that hordein transport to the PSV at least partially bypasses the Golgi body and involves autophagy or an autophagy-like processes. The *in vivo* observation of loose TIP3-GFP-labelled (and MDY-64-stained) membranes and vesicles within TIP3-GFP-positive PSVs in the barley subaleurone lends further support to this hypothesis. In wheat, it is well established that prolamin bodies enriched in gliadins aggregate downstream of the Golgi body, whereas prolamin bodies enriched in glutenins form in the ER and are then sequestered into PSVs by an autophagy-like process (Levanony *et al.*, 1992; Reyes *et al.*, 2011). In addition, Reyes *et al.* (2011) reported that zeins are delivered to aleurone PSVs in atypical prevacuolar compartments that may arise at least partially by autophagy. Alternatively, the presence of ER membranes within the PSVs may indicate a mechanism involving the budding of membrane-containing vesicles from the ER. These would function as prevacuolar organelles and deliver membrane arrays to the PSVs (Rogers, 2011).

Some protein bodies in the central starchy endosperm (12 DAP onwards) lacked complete TIP3-GFP-labelled membranes, which appeared to degenerate during seed maturation. When considered together with the collapsing subaleurone PSVs at the same developmental stage, it seems that the integrity of TIP3-GFP-labelled membranes in the starchy endosperm declined during maturation, in contrast to the strong and continuous TIP3-GFP signal and lack of morphological changes in aleurone PSVs. Unlike the starchy endosperm, the aleurone layer is protected against desiccation-induced injury (Stacy *et al.*, 1999). Therefore, it seems likely that desiccation during endosperm development may contribute to the dynamic behaviour of the membranes. Indeed, it has been shown that dehydration during maturation has a physical impact on membrane lipids, including demixing and fluid-to-gel phase transitions (Bryant *et al.*, 2001). Moreover, water loss during maturation causes molecular crowding, which increases the viscosity of the cytosol, thus promoting interactions that cause protein denaturation and membrane fusion (Hoekstra *et al.*, 2001). Seed dehydration also induces the uncontrolled production of reactive oxygen species, which may cause the peroxidation and de-esterification of membrane lipids (Hoekstra *et al.*, 2001; Sattler *et al.*, 2006; Tang *et al.*, 2012). This in turn leads to the irreversible formation of gel-phase domains and the loss of membrane function (McKersie *et al.*, 1990). The accumulation of nonenzymic lipid peroxidation products can disrupt the selective permeability of the lipid bilayer and cause the leakage of electrolytes and small molecules, including water (Keppler and Novacky, 1986; Sattler *et al.*, 2006; Onda *et al.*, 2009). Further investigations are needed to characterize the molecular nature of membranes during endosperm development and to investigate possible associations between desiccation, membrane stress, and endomembrane reshaping.

In conclusion, this study investigated the fate of TIP3-GFP-labelled PSVs during barley endosperm development and revealed cell-layer-specific differences in morphological changes related to the developmental stage and cell age. The TIP3-labelled PSVs of the subaleurone at 10 DAP are characterized by dynamic reshaping events that are not so profound in the other layers. In the maturing starchy endosperm, the protein bodies are no longer fully surrounded by TIP3-GFP-labelled membranes, indicating that PSV membrane degeneration occurs in later development, probably as a consequence of desiccation.

## Supplementary material

Supplementary data are available at *JXB* online.

Supplementary Fig. S1. Bright-field images corresponding to the fluorescence images in Fig. 1 (A) and histochemical analysis of TIP3-GFP in the aleurone and subaleurone (B).

Supplementary Fig. S2. Transmission electron micrographs of barley endosperm cells shows membranes closely surrounding the protein bodies.

Supplementary Movie M1. Movie corresponding to Fig. 5.

Supplementary Data
