# Supplementary material for: Fusion, rupture, and degeneration: the fate of in vivo-labelled PSVs in developing barley endosperm
Source: J Exp Bot. 2014 May 6;65(12):3249–61. doi: 10.1093/jxb/eru175 (PMC4071841; doi:10.1093/jxb/eru175)
Supplement: Supplementary Data [file supp_65_12_3249__index.html]

Fusion, rupture, and degeneration: the fate of in vivo-labelled PSVs in developing barley endosperm — Fusion, rupture, and degeneration: the fate of in vivo-labelled PSVs in developing barley endosperm — Supplementary Data 

# Fusion, rupture, and degeneration: the fate of *in vivo*-labelled PSVs in developing barley endosperm

## Supplementary Data

Data files

**Files in this Data Supplement:**

- Supplementary Data - Supplementary Data
- Supplementary Data - Supplementary Data
