# Supplementary material for: Fusion, rupture, and degeneration: the fate of in vivo-labelled PSVs in developing barley endosperm
Source: J Exp Bot. 2014 May 6;65(12):3249–61. doi: 10.1093/jxb/eru175 (PMC4071841; doi:10.1093/jxb/eru175)
Supplement: Supplementary Data [file supp_eru175_jexbot118224_file001.pdf]

Supplementary Material

Fig. S1. A. Bright field images corresponding to the fluorescence images in Fig 1. B. Histochemical analysis of TIP3-GFP in the aleurone (asterisk) and subaleurone. In the aleurone, TIP3-GFP also weakly labels the plasma membrane (arrowheads). C. Sections of fixed barley endosperm treated with toluidin blue are shown for comparison. Putative PSVs are visible in the aleurone (asterisk), the protein content of the PSVs in subaleurone and starchy endosperm is stained in blue (arrowheads) and putative vacuolar compartments are indicated in the starchy endosperm (black arrows). The white arrows in the overview indicate the subaleurone and the starchy endosperm, respectively. Scale bar = 5 µm.

Fig. S2. Transmission electron micrographs of barley endosperm cells show membranes (arrows) closely surrounding the protein bodies (asterisks). Scale bar = 1 µm.

M1. Movie corresponding to figure 5.

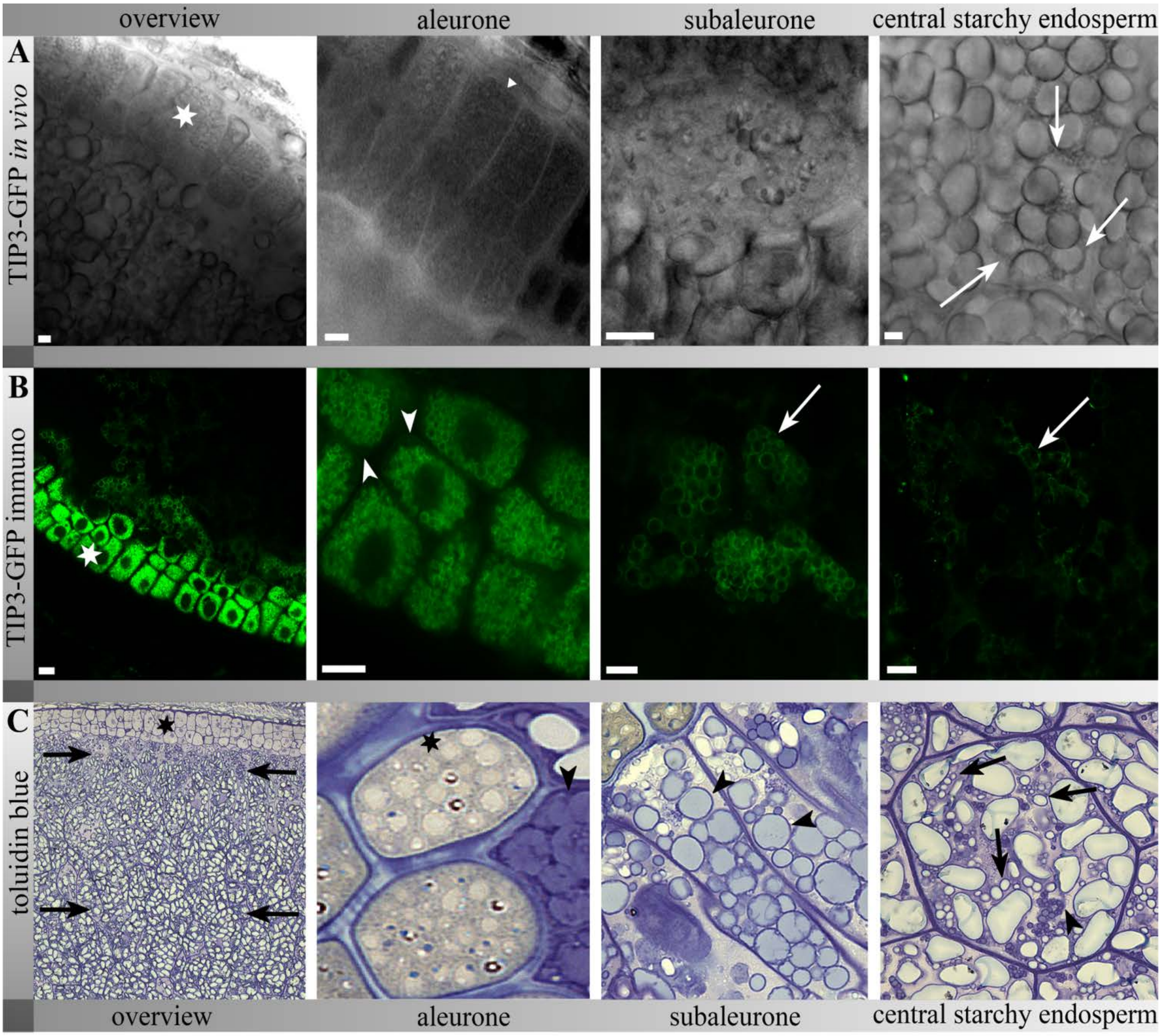
overview
aleurone
subaleurone
central starchy endosperm
A
TIP3-GFP in vivo
B
TIP3-GFP immuno
C
toluidin blue
overview
aleurone
subaleurone
central starchy endosperm

Figure S1

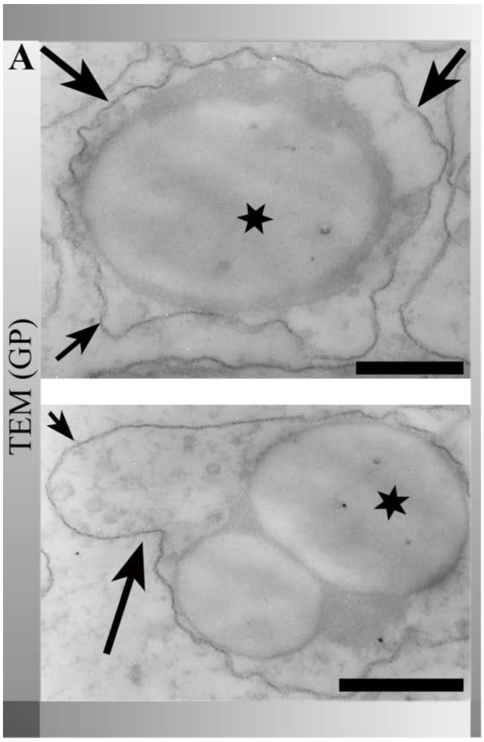

Figure S2
